# ASK FOR IT: An Internet-based educational program for patients with atrial fibrillation – Results from a pilot study and design of the randomized, controlled, multicenter ASK FOR IT study

**DOI:** 10.1016/j.cvdhj.2020.11.003

**Published:** 2020-11-13

**Authors:** Ulla Walfridsson, David Brohede, Håkan Walfridsson

**Affiliations:** ∗ Department of Cardiology, University Hospital, and Department of Medical and Health Sciences, Linköping University, Linköping, Sweden; † Clinical and Digital Psychology, Psykologpartners W&W AB, Linköping, Sweden

**Keywords:** Atrial fibrillation, Disease-specific questionnaire, Health-related quality of life, Internet-based education, Structured care, Symptoms

## Abstract

**Background:**

In the structured care of patients with atrial fibrillation (AF), education is compulsory. Patients search for information but sources of reliable information are sparse. ASK FOR IT, an internet- and guideline-based educational program, offers such information.

**Objective:**

To describe the development of ASK FOR IT, report on a pilot study, and present the design of a randomized controlled trial evaluating the benefits of ASK FOR IT in addition to standard care on symptoms, health-related quality of life (HRQoL), and health economy.

**Methods:**

ASK FOR IT was developed by healthcare providers, patients, and a psychologist. ASK FOR IT contains 6 parts: basic mechanisms, symptoms, treatment options, diagnostic possibilities, lifestyle management, and a mental support section. The following questionnaires were used: SF-36, EQ-5D, the disease-specific ASTA (symptoms and HRQoL), and HADS (depression and anxiety). Interviews regarding usability and understanding were conducted.

**Results:**

Pilot study: Fifteen patients (mean age 65 years), 4 women and 11 men, took part in the study. During follow-up, the patients improved regarding symptoms in ASTA (*P* = .038) and the HRQoL mental domain (*P* = .011), while no differences were seen in SF-36, EQ-5D, or HADS. Interviews indicated that the program was easy to use and the content easy to understand.

**Conclusion:**

The ASK FOR IT program functioned as intended. It was easy to use and the information was easy to understand. The significant reduction in symptoms and improvement in HRQoL (mental domain) after only 3 months are encouraging. In the main study, 200 patients will be randomized.

## Graphical abstract

**Figure:**
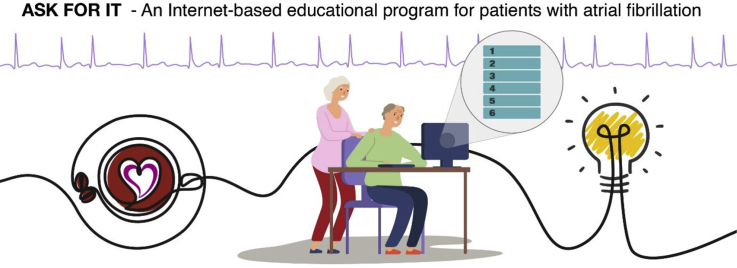

**Key Findings**

• The significant reduction in symptoms and improvement in mental health-related quality of life (HRQoL) after working with the ASK FOR IT program for 3 months are encouraging, as symptom reduction and HRQoL are important aspects in the care.
• Education is compulsory in the care of patients with atrial fibrillation (AF) and ASK FOR IT provides easily accessible information for many patients. It was valuable to include the experts themselves (ie, patients with AF) during the development of the program, with education aimed for them.
• Regardless of age, the ASK FOR IT program was easy to use, the content was found to be informative and easy to understand, and ASK FOR IT provided important help to handle AF and AF episodes.

**Snapshot summary**

ASK FOR IT is an internet-based educational program developed for patients with atrial fibrillation. The paper presents data from a pilot study and the design of a randomized controlled study. The primary endpoint is symptom reduction.

## Introduction

Modern care of patients with atrial fibrillation (AF) focuses on stroke prevention, on elimination or at least reduction of symptoms, and on the impact of AF on health-related quality of life (HRQoL).^1^ The important study by Hendriks and colleagues^2^^,^^3^ showed a reduction in hospitalization and in mortality from a structured care program including patient education. The guidelines regarding treatment of patients with AF underline the importance of patient education.^1^^,^^4^

ASK FOR IT is an internet-based educational program developed for patients with AF, containing 6 different parts with information on basic mechanisms, symptoms, treatment, diagnostic possibilities, lifestyle management, and a mental support section. The hypothesis to be tested is that well-educated patients will experience fewer symptoms and less negative impact on HRQoL, will feel more secure, and will consume less medical care. The aim of this paper is to present the development of the ASK FOR IT educational program and to present data from a pilot study and the design of a prospective, randomized study (RCT).

## Methods

### Development of the internet-based educational program ASK FOR IT

#### Interviews

During the development and validation of the arrhythmia-specific questionnaire ASTA,^5^^,^^6^ patients often asked for information about their AF condition and wanted such information to be easily accessible, specifically between visits. To enable the provision of trustworthy guideline-based information, the ASK FOR IT program was developed. Since it is well known that some patients are in extra need of mental support, a collaboration was initiated with a psychologist skilled in cognitive behavioral therapy (CBT) and in building programs for internet-based information. During a period of 4 months, the first author (U.W.) conducted interviews with healthcare personnel working with patients with AF and patients in different stages of treatment of their arrhythmia. The interview guide consisted of an open-ended question: “What would you like to include in an education program that deals with information about arrhythmia/AF?” aiming for a better understanding of the degree of knowledge present in our patient population. Although there were considerable differences between patients, knowledge was generally very limited. A number of areas were identified and included in ASK FOR IT.

#### Checklist

The first author (U.W.), in collaboration with Prof. Jeroen Hendriks (Adelaide, Australia), created a checklist to hand out to patients coming either for DC conversion or catheter ablation (CA)—26 and 16 patients, respectively—to investigate what the patients wanted to know about AF. The age of the 42 patients ranged between 46 and 90 years, and 19 were women (45%). The questions were based on information from the interviews, literature, and long-term experiences of working with patients with AF. The questions concerned what they wanted information about, how they wanted the information, when they found themselves in most need of information, whether they had access to the internet, what kind of information they already had, and what they found missing in that information. The feedback resulted in an extensive document covering everything from “What is AF?” to “How can I handle my symptoms and anxiety in connection with AF?”

The data led to a 6-step program including basic mechanisms, symptoms, treatment and diagnostic possibilities, lifestyle management, and mental support.

### Presentation of the educational program ASK FOR IT

#### Overview of the program

ASK FOR IT is an online educational program for patients with AF. The program consists of 6 separate steps, requiring approximately 6 hours when studied carefully, including answering questions at the end of each step. The questions relate to the current step and are aimed to explore the patient’s own situation. The patients are recommended to go slowly through the 6 parts and to complete the program in no sooner than 6–8 weeks (ie, 1 step per week).

#### Content of the steps

Each step (module) presents research-based knowledge about AF, its possible causes, diagnostics, treatment, and self-care measures. Links to official, validated medical information are provided throughout the program, enabling further optional reading about the mentioned drugs. Some steps contain animations describing heart function, sinus rhythm, AF, and different treatments.

Table 1 presents an overview of the content and exercises.

**Table 1:** Content overview

Module/Step	Content	Exercises
1: Mechanism of AF	Basic knowledge about the well-functioning heart, ECG, AF, symptoms, influence on daily life concerns and possible causes.	Mapping out personal symptoms and life impact.
2: Tests and diagnostics	Information about the most common diagnostic tools and their procedures. Introduction to diagnostic scales such as CHA_2_DS_2_VASc and EHRA scores.	CHA_2_DS_2_VASc estimation, BMI scoring, personal experiences from debut, diagnostics and previous treatment.
3: Treatments of AF: drugs and cardioversion	Overview of medication options and cardioversion.	Previous medication, side effects, and planned follow-up.
4: Treatments of AF: ablation and other treatments	Overview of treatment principles in ablation and other options.	Previous treatments including ablation pacemaker and surgical treatments. Side effects and planned follow-up.
5: The significance of lifestyle	Factors influencing impact severity, including weight loss, sleep, tobacco use, and other possible self-help actions.	Personal goal-setting concerning lifestyle.
6: Living with AF	Research-based information on experiences of AF's impact on everyday life. Acceptance- and commitment-inspired focus on acceptance of factors outside the patient's control and commitment to choices within the patient's control.	Mapping of strategies used by the patient to cope with the situation, including selection of constructive coping strategies. Evaluation of the program itself.

AF = atrial fibrillation; BMI = body mass index; EHRA = European Heart Rhythm Association.

There is a patient case (speaker voice) related to the content in each step, telling a story about a person suffering from AF.

Each step comprises 7500–18,000 characters, not including animated videos.

#### Content development and instructional design

The aim of the project was to design a scientifically valid educational program for patients with AF. Therefore, the focus was on presenting scientifically confirmed information in a pedagogical way.

The basic content was laid out by the first author of this article. This body of information was based on clinical expertise and previous research. The instructional designer, a psychologist specialized in online learning, used design principles gathered from cognitive psychology, neuroscience, and previous experience to develop and refine the overall structure and content format.^7^ Inductive methods were used by the instructional designer to categorize, reorder, and clarify the information. Higher-order structure, flow, and distribution were looked over. Additional content, exercises, and headlines were added. The overall aim was to aid the patient in increasing self-help/self-management strategies. The language was adapted to better suit a nonacademic audience. Finally, instructional media was produced to illustrate complex concepts and treatments. The result was a clear, concise, and appealing content, where text, photographs, and animations complemented each other.

#### Delivery and technology

ASK FOR IT is delivered through a platform otherwise used for internet-based CBT. Access requires 2-factor authentication: upon login the patient uses his or her personal username and password and the system then generates a 6-digit text message that is sent to the patient’s smartphone. All data, including personal data, exercise answers, and activity log, are stored in an encrypted database, with medical-grade safety measures for both data and physical safety.

A dedicated database, based on guidelines for AF care, was used. It served as a checklist to explore what investigations were performed and what should be considered, the chosen treatment strategy (eg, rhythm vs rate control regime), and lifestyle concerns such as physical activities, smoking, and alcohol habits.

### Pre and pilot study of the ASK FOR IT program

#### Focus group discussions

When the initial development and IT platform building phase was completed, a focus group pretested the program. Five persons with AF, 2 women and 3 men, aged 43–72 and being treated at the University Hospital in Linköping, were invited to take part in the focus group. Some had been through repeated CA procedures while some were at the initial phase of living with AF, eg, they had just been diagnosed with AF, or they were on antiarrhythmic medication and/or had experiences of DC conversion.

The 3 authors met the patients twice at the University Hospital. The authors were the nurse (U.W.) primarily responsible for the development of the ASK FOR IT program, the electrophysiologist (H.W.) included in creating the educational program, and the psychologist (D.B.) skilled in CBT and responsible for building the program on an internet platform.

At the first meeting the patients were given a presentation of the program where each part of ASK FOR IT was discussed in detail. The patients were informed about how to work with the program in order to be available to evaluate the content, readability, and understandability. At the second meeting the patients gave feedback on the program. Some modifications to the educational program were suggested and incorporated in ASK FOR IT, such as changes in some headings, moving some paragraphs to a more relevant place, and some extended explanations regarding the interventions. After the program was updated, a pilot study was performed.

### Phase 1: The ASK FOR IT pilot study

#### Introduction

In order to evaluate ASK FOR IT concerning symptoms, HRQoL, and symptoms of anxiety and depression, a pilot study was conducted to test logistics and feasibility for a prospective, randomized study. The ASTA 9-item symptom scale score in the pilot study was to be the basis for the power calculation.

#### Methods

##### Informed consent and ethical considerations

The Regional Ethical Review Board at the Faculty of Health Sciences, Linköping, Sweden (DNR 2016-349-31) approved the study. Each patient provided written informed consent in the pilot and RCT study. The study complies with the Declaration of Helsinki.^8^

##### Study patients

The study team made a convenient sample of patients with AF in order to test ASK FOR IT on a small but heterogeneous sample. Fifteen patients were asked to participate, 4 women and 11 men, ranging in age from 39 to 82 years. Some had a short history of AF; others had a moderately long or very long history, including after previous CA (Table 2). AF history varied from only months to 34 years, with a mean of 9.6 years (Table 3). Patients invited to participate were those with a planned visit at the hospital’s outpatient clinic. The patients were contacted by the first author (U.W.) and were sent written study information.

**Table 2:** Patient characteristics

Number of patients	15
Age (years), mean (range)	64.7 (39–82)
Sex (women)	4
Sex (men)	11
Body mass index (kg/m^2^), mean (± SD)	27.8 (± 4.5)
Paroxysmal AF	9
Persistent AF	4
Permanent AF	2
Congestive heart failure	0
Hypertension	7
Diabetes	2
TIA/stroke	1
Vascular disease	1
Ischemic heart disease	1
CHA_2_DS_2_VASc	
0	5
1	2
2	1
3	3
4	4
5	0
6	0
7	0
8	0
Antiarrhythmic drugs	
Beta blockers	10
Calcium antagonists	0
Digitalis	0
Class 1A	0
Class 1C	1
Class III sotalol	0
Amiodarone	3
Dronedarone	0
Anticoagulants	
Warfarin	5
NOAC	7

NOAC = new oral anticoagulants; TIA = transient ischemic attack.

Class IA and IC: Classification according to Vaughan Williams for antiarrhythmic drugs; no patient was on calcium antagonists, digitalis, class IA, sotalol, or dronedarone.

**Table 3:** Details on each participating patient

	Age	Sex	AF history (years)	AF type	EHRA score	BMI	CHA_2_DS_2_VASc	Congestive heart failure	Hypertension	Diabetes	Vascular disease	Ischemic heart disease	TIA/stroke	β-blocker	Class 1C	Amiodarone	Warfarin	NOAG
Patient 1	82	M	10	Permanent	2b	34	4	0	X	0	X	0	0	X	0	0	X	0
Patient 2	80	M	9	Permanent	3	28	3	0	X	0	0	0	0	X	0	0	X	0
Patient 3	79	M	34	Paroxysmal	2b	25	4	0	X	0	0	X	0	0	0	X	X	0
Patient 4	79	F	2	Persistent	4	24	3	0	0	0	0	0	0	X	0	X	0	X
Patient 5	75	F	9	Paroxysmal	3	32	4	0	X	0	0	0	0	X	0	0	X	0
Patient 6∗	73	F	9	Persistent	3	27	4	0	X	X	0	0	0	X	0	0	X	0
Patient 7	65	M	12	Paroxysmal	3	31	1	0	0	0	0	0	0	0	0	0	0	X
Patient 8	63	M	7	Paroxysmal	1	24	2	0	X	X	0	0	0	0	0	0	0	X
Patient 9	60	M	2	Paroxysmal	2b	31	0	0	0	0	0	0	0	X	X	0	0	X
Patient 10	59	M	10	Persistent	3	24	3	0	X	0	0	0	X	X	0	X	0	X
Patient 11	57	F	14	Paroxysmal	3	37	1	0	0	0	0	0	0	0	0	0	0	X
Patient 12	55	M	2	Paroxysmal	3	26	0	0	0	0	0	0	0	X	0	0	0	0
Patient 13	55	M	<1	Paroxysmal	3	25	0	0	0	0	0	0	0	X	0	0	0	0
Patient 14	50	M	<1	Persistent	4	27	0	0	0	0	0	0	0	X	0	0	0	X
Patient 15	39	M	20	Paroxysmal	3	22	0	0	0	0	0	0	0	0	0	0	0	0

AF = atrial fibrillation; BMI = body mass index; EHRA = European Heart Rhythm Association; NOAC = new oral anticoagulants; TIA = transient ischemic attack.

Class IA and IC: Classification according to Vaughan Williams for antiarrhythmic drugs; no patient was on calcium antagonists, digitalis, class IA, sotalol, or dronedarone.

∗ This patient did not complete the study.

### Study outcomes

#### Patient-reported outcome measures

##### The Medical Outcomes Study 36-Item Short Form Health Survey

The “Medical Outcomes Study 36-Item Short Form Health Survey” (SF-36) is a generic quality-of-life questionnaire comprising 36 items, of which 35 evaluate 8 scales ranging from 0 (worst possible health) to 100 (best possible health).^9^ The 8 scales are summarized in physical and mental component summaries, standardized to a norm with a mean of 50 and a standard deviation (SD) of 10.^10^

##### EuroQuol 5 dimensions

The “EuroQuol” 5 dimensions, 5 levels (EQ-5D-5L) is an instrument that evaluates generic quality of life. It was developed in Europe and is widely used. The EQ-5D-5L descriptive system is a preference-based HRQoL measure with a 5-point response scale and 1 question for each of the 5 dimensions: mobility, self-care, usual activities, pain/discomfort, and anxiety/depression. There is also a visual analogue scale recording the respondents' self-rated health status.^11^

##### The arrhythmia-specific questionnaire in tachycardia and arrhythmia

The disease-specific “arrhythmia-specific questionnaire in tachycardia and arrhythmia” (ASTA) is a validated questionnaire and has been previously described in detail.^5^^,^^6^^,^^12^ In brief, 9 items assess symptom burden (ASTA symptom scale) and 13 items assess HRQoL (ASTA HRQoL scale), all with a 4-point response scale (Tables 4 and 5). The questionnaire was used in its web version. The scale scores range from 0 to 100, where higher scores reflect a higher symptom burden and a worse effect on HRQoL because of the arrhythmia. Results from a large cohort of patients with AF have been published.^13^

**Table 4:** The 9 individual items in the ASTA symptom scale

Item	Symptom
1	Breathlessness during activity
2	Breathlessness even at rest
3	Dizziness
4	Cold sweats
5	Weakness/fatigue
6	Tiredness
7	Chest pain
8	Pressure/discomfort in chest
9	Worry/anxiety

**Table 5:** The 13 individual items in the ASTA health-related quality-of-life scale

Item	Subscale
Do you feel unable to work, study or carry out daily activities as you would like to due to your arrhythmia?	Physical
Do you spend less time with your family/relatives and friends than you would like to due to your arrhythmia?	Physical
Do you spend less time with acquaintances (people you do not know that well) than you would like to due to your arrhythmia?	Physical
Do you avoid planning things you would like to do, for instance travelling or leisure activities due to your arrhythmia?	Physical
Is your physical ability impaired due to your arrhythmia?	Physical
Is your ability to concentrate impaired due to your arrhythmia?	Mental
Do you feel dejected or sad due to your arrhythmia?	Mental
Do you feel irritated or angry due to your arrhythmia?	Mental
Do you experience sleep problems due to your arrhythmia?	Mental
Is your sexual life affected negatively by your arrhythmia?	Physical
Are you afraid of dying due to your arrhythmia?	Mental
Has your life situation deteriorated due to your arrhythmia?	Physical
Do you feel worried that your symptoms will re-occur during the periods when you do not have arrhythmia?	Mental

##### The Hospital Anxiety and Depression Scale

The domain-specific “Hospital Anxiety and Depression Scale” (HADS) questionnaire consists of 2 subscales, where 7 items assess anxiety and 7 assess depression. The score for each subscale ranges from 0 to 21. The scores are categorized as normal (0–7), possible (8–10), and probable (≥11) anxiety and depression, respectively.^14^

The patients also answered questions regarding knowledge about AF (ie, disease-related questions).

#### Interviews

The patients were interviewed about healthcare consumption related to AF (an extended description is included later regarding the RCT) and about their experiences of working with the ASK FOR IT program.

Three study visits were performed: at baseline, when seeing an electrophysiologist (H.W.) and signing the informed consent; after a week, with the study nurse to begin the ASK FOR IT program and to check that the ingoing questionnaires had been filled out; and at the last study visit after 3 months.

The arrhythmia-specific ASTA questionnaire was used as a basis for evaluation of the patient’s arrhythmia-related situation at the visit. During the 3-month period the study nurse (U.W.) followed the patients’ work in the program on a weekly basis.

At baseline and at the 3-month follow-up visit, electrocardiogram (ECG), weight, and blood pressure were checked; at baseline, height (body mass index) and blood samples were collected.

### Statistical analysis

Continuous variables are expressed as means ± SD. Categorical data are presented as counts and percentages. A paired sample *t* test was used for comparisons over time regarding the outcome of the study questionnaires. Effect sizes (ES) were calculated for estimation of the significant differences in the arrhythmia-specific ASTA questionnaire using Cohens’ d, where 0.2 was a small ES, 0.5 was moderate, and 0.8 was large.^15^ All reported *P* values are 2-sided and a value <.05 is considered statistically significant.

## Results

Fourteen of the 15 recruited patients with a diagnosis of paroxysmal/persistent or permanent AF, mean age 65 years, completed the 3-month follow-up study. One of the 4 women withdrew from the study owing to difficulties with her internet connection, making it impossible to work with ASK FOR IT.

Details on comorbidities and medication for each patient are presented in Table 3.

### ASTA

The results of the ASTA symptom scale scores and HRQoL scale scores are presented in Table 6. Two significant changes were observed over time. During the 3-month follow-up period, patients improved regarding symptoms where the ASTA symptom scale score was lower; that is, the patients experienced fewer symptoms (*P* = .038), with a moderate ES for the changes over time (0.62), and an improvement was seen in the ASTA HRQoL mental domain scale scores (*P* = .011), also with a moderate ES (0.79).

**Table 6:** Overall scoring in the ASTA symptom and health-related quality-of-life scales

	Mean (baseline)	SD (baseline)	Mean (3 months)	SD (3 months)	Sig (2-tailed) (3 months)	Cohen’s d (ES)∗
Symptom score 9 items	31.93	9.98	21.43	15.01	.038	0.62
HRQoL total score 13 items	26.71	17.95	20.21	17.06	.189	
Physical score 7 items	28.93	22.52	23.57	25.30	.440	
Mental score 6 items	23.50	14.83	14.79	9.93	.011	0.79

ES = effect size; HRQoL = health-related quality of life; Sig = significance.

∗ Small 0.2, medium 0.5, large 0.8.

### SF-36

No differences were found in 8 SF-36 scales or in the physical (*P* = .439) or mental (*P* = .993) domains between baseline and the 3-month follow-up.

### EQ-5D

No differences over time were found in EQ-5D, nor in the EQ-5D-index and EQ visual analogue scale (*P* = .370, *P* = .062, respectively).

### HADS

The HADS questionnaire, exploring anxiety and depression, did not show any differences over time (anxiety *P* = .907, depression *P* = .089).

### Interviews

In this pilot study, some outcomes were important to focus on regarding logistics, the content of the program, and how to navigate within the program. The interviews showed that regardless of age, the program was easy to use and the content was found to be informative and easy to understand. Regarding logistics from a healthcare provider perspective, it was shown that there was a need for support and guidance, particularly to help patients get started with the ASK FOR IT program.

In the last step in the program (step 6) the patients summarized the experience of working with ASK FOR IT: “Knowledge provided security,” “An insight of not being the only one with AF,” and that “ASK FOR IT provided important help to handle AF and AF episodes.”

## Discussion

### Pilot study

The main findings from the pilot study were the significant reduction in arrhythmia-related symptoms (this is the primary endpoint in the prospective main study) and the positive effect seen regarding arrhythmia-related impact on the mental part of HRQoL, while no significant changes were seen in the generic or domain-specific instruments. The internet-based ASK FOR IT program functioned as was intended, in that patients found it easy to navigate and found that the content was informative and easy to understand. Regarding logistics concerns, some patients needed support to get started, others with saving the text for their responses or when having problems to print out pdf files. The patient could send a message to the study nurse within the program or to the support department responsible for the information technology platform and ask for help if needed. When a patient sent a message, the study nurse received an automatic e-mail highlighting that there was a message from a patient in the ASK FOR IT study.

During the interviews the patients made many positive comments about the program. They appreciated getting a more complete picture of AF and they felt more secure, realizing that they were not alone in this situation, and they found it valuable to get hints on how to handle different issues related to AF. The content was easy to understand, and the usability (ie, how to navigate) was intuitive. This also applied to the links and the animations in the ASK FOR IT program.

There was a request for access to links with extended information on AF—for example, links to scientific publications. This is included in plans for further development of the ASK FOR IT program after the RCT is finished.

Some of the patients were a little anxious about their capability to work with an internet-based program but were willing to try. A few patients sometimes had difficulties finding time for ASK FOR IT after a long working day or because they lived with children who wanted attention in the evenings.

In the latest updated guidelines for 2020 there are recommendations to include assessment of symptoms and daily life concerns in the care by using patient-reported outcome measures.^4^ In this project the arrhythmia-specific ASTA forms the basis for the discussions during visits, enabling to focus on what impact AF has on the patient’s daily life regarding symptoms and various aspects of HRQoL.

### Methodological considerations/limitations

We found that there was a need to check, on a weekly basis, that patients were really working on the program and their homework with questions after each step in the program. One patient struggled with the internet connection at home and never started working on the program, and another patient had difficulties saving the responses to the homework, and needed some support.

The study nurse was the only one who could see what the patient had written. The routine for the study nurse was to, on a weekly basis, log in to the program and check if the patient had worked with the program and responded to the questions after each step, so as to evaluate the patient’s progress. The time the nurse spent with this was approximately 30 minutes per week.

Obvious limitations of ASK FOR IT are the need for internet access as well as the fact that ASK FOR IT is only available in Swedish at present.

## Conclusion

The internet-based ASK FOR IT program functioned as intended. Patients found it easy to use and the information easy to understand. The observed significant reduction in symptoms and particularly the improvement in HRQoL (mental domain) after only 3 months in such a small sample is encouraging.

### Phase 2 Study design of the randomized, controlled, multicenter ASK FOR IT study

**Figure 1:**
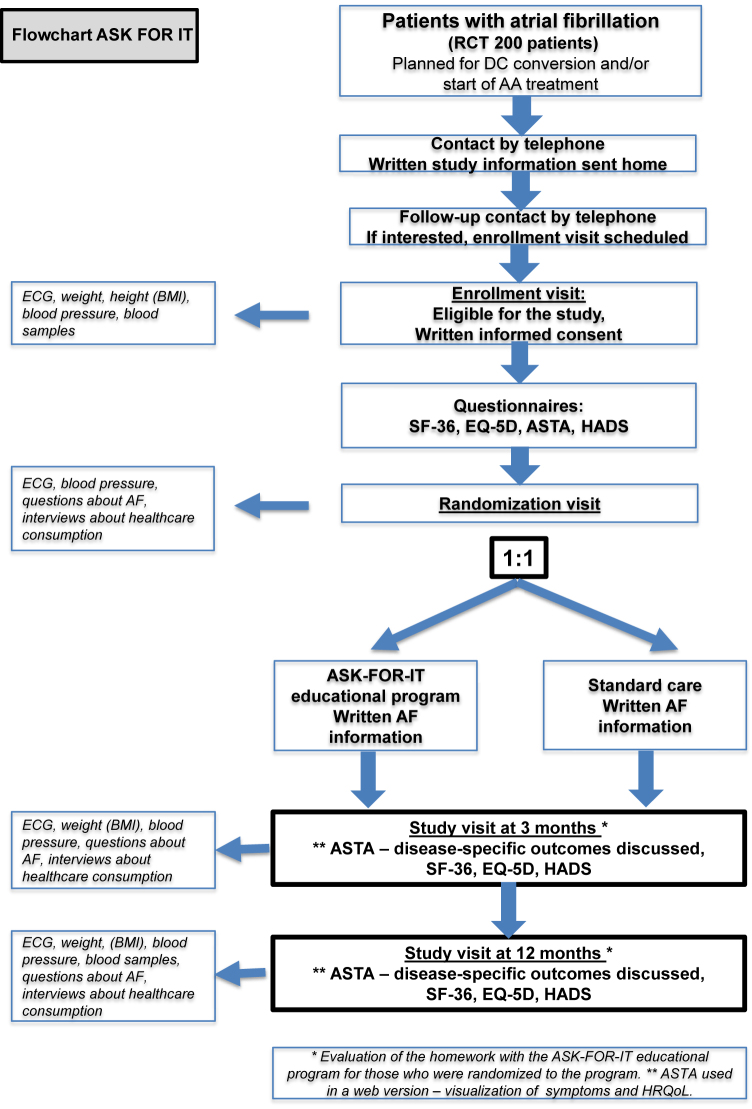
Flow chart for the prospective, randomized control study ASK FOR IT. AA treatment = antiarrhythmic medication; AF = atrial fibrillation; ASTA = arrhythmia-specific questionnaire in tachycardia and arrhythmia; BMI = body mass index; ECG = electrocardiogram; EQ-5D = EuroQuol 5 dimensions; HADS = Hospital Anxiety and Depression Scale; HRQoL = health-related quality of life; RCT = randomized controlled study; SF-36 = The Medical Outcomes Study 36-Item Short Form Health Survey.

The ASK FOR IT study is a prospective, randomized, controlled, parallel, multicenter study. Eligibility criteria are as follows: patients ≥18 years; with paroxysmal or persistent AF scheduled for DC conversion and/or treatment with antiarrhythmic drugs; having sufficient knowledge of the Swedish language and capable of independently filling out the questionnaires and completing the program; having a connection to the internet (computer/iPad); with no medical conditions making it troublesome to fulfill the 12-month study period; not scheduled for heart surgery or CA at the time of enrollment/randomization; and willing to participate. Patients are randomly assigned either to receive the internet-based educational program ASK FOR IT or to receive standard care with extra written information added about AF in a 1:1 fashion. The first patient was randomized in November 2018. A total of 200 patients will be included and followed for a period of 12 months after inclusion. As of August 2020, approximately 100 patients had been randomized; enrollment is expected to be completed by July 2021 (flow chart, Figure 1).

#### Study patients

Study patients are recruited from 4 centers in Sweden: Linköping University Hospital, Örebro University Hospital, the Regional Hospital Ryhov in Jönköping, and the Sahlgrenska University Hospital in Mölndal. Patients who meet the eligibility criteria and have none of the exclusion criteria will be recruited by a cardiologist/electrophysiologist at each enrolling center. Reasons for exclusion are recorded for eligible patients who are not enrolled.

The patients have a full baseline evaluation performed, including medical history, a physical examination, a 12-lead ECG, weight, height (body mass index), blood pressure, and blood samples (including NT-proBNP, hs-CRP), to be repeated at the 12-month follow-up visit.

The questionnaires to be filled out are SF-36, EQ-5D, ASTA, and HADS (described earlier), and a questionnaire to explore patients’ knowledge of AF.

#### Health economy

The use of healthcare owing to arrhythmia-related issues during the 12 months prior to randomization and during the 12-month study period is documented through interviews and available medical records. The following will be recorded: number of visits to the emergency room, number of visits to primary care units and hospital outpatient clinics, and number of days spent in hospital. The costs for each such visit will be calculated. The estimated consumption of prescribed medication will also be recorded. Since most primary care centers are connected to the same administrative network as the hospital, this can be done with a high degree of accuracy. The patients are also asked if they have sought private medical care owing to arrhythmia-related concerns.

#### Randomization

Block randomization is used with stratification for center and sex. Envelopes containing sealed information on randomization group for each patient are prepared by “Forum Östergötland – Linköping University” and stored at each participating center. Patients are randomized to either (1) work with the ASK FOR IT program or (2) care as usual but obtaining extra written information (brochures).

#### Evaluation after 3 and 12 months

Parameters registered at baseline will be repeated after 3 and 12 months—ie, medical history, physical examination, 12-lead ECG, weight, blood pressure, and blood samples (at baseline and at 12 months). The questionnaires SF-36, EQ-5D, ASTA, and HADS and questions regarding knowledge about AF are again filled out. The use of healthcare is recorded as previously mentioned, and at 3 and 12 months the patients are asked about their experiences of working with the ASK FOR IT program.

#### Power calculation

Based on the results of the pilot study on improvement in the ASTA 9-item symptom scale score (baseline 31.93 and at 3 months 21.43; Table 6) and with an expected improvement of 5 scores/points (SD 11.7) based on data from 188 patients treated with DC conversion, we calculate that with a power goal of 80% and type I error of 0.05 there is a need to include 182 patients in the RCT study. In order to secure an adequate number of evaluable patients, considering some drop-outs, a decision was reached to aim for 200 randomized patients. The analyses are performed using SPSS 25.0 (SPSS, Chicago, IL).
